# CovPDB: a high-resolution coverage of the covalent protein–ligand interactome

**DOI:** 10.1093/nar/gkab868

**Published:** 2021-09-28

**Authors:** Mingjie Gao, Aurélien F A Moumbock, Ammar Qaseem, Qianqing Xu, Stefan Günther

**Affiliations:** Institute of Pharmaceutical Sciences, Albert-Ludwigs-Universität Freiburg, Hermann-Herder-Straße 9, D-79104 Freiburg, Germany; Institute of Pharmaceutical Sciences, Albert-Ludwigs-Universität Freiburg, Hermann-Herder-Straße 9, D-79104 Freiburg, Germany; Institute of Pharmaceutical Sciences, Albert-Ludwigs-Universität Freiburg, Hermann-Herder-Straße 9, D-79104 Freiburg, Germany; Institute of Pharmaceutical Sciences, Albert-Ludwigs-Universität Freiburg, Hermann-Herder-Straße 9, D-79104 Freiburg, Germany; Institute of Pharmaceutical Sciences, Albert-Ludwigs-Universität Freiburg, Hermann-Herder-Straße 9, D-79104 Freiburg, Germany

## Abstract

In recent years, the drug discovery paradigm has shifted toward compounds that covalently modify disease-associated target proteins, because they tend to possess high potency, selectivity, and duration of action. The rational design of novel targeted covalent inhibitors (TCIs) typically starts from resolved macromolecular structures of target proteins in their apo or holo forms. However, the existing TCI databases contain only a paucity of covalent protein–ligand (cP–L) complexes. Herein, we report CovPDB, the first database solely dedicated to high-resolution cocrystal structures of biologically relevant cP–L complexes, curated from the Protein Data Bank. For these curated complexes, the chemical structures and warheads of pre-reactive electrophilic ligands as well as the covalent bonding mechanisms to their target proteins were expertly manually annotated. Totally, CovPDB contains 733 proteins and 1,501 ligands, relating to 2,294 cP–L complexes, 93 reactive warheads, 14 targetable residues, and 21 covalent mechanisms. Users are provided with an intuitive and interactive web interface that allows multiple search and browsing options to explore the covalent interactome at a molecular level in order to develop novel TCIs. CovPDB is freely accessible at http://www.pharmbioinf.uni-freiburg.de/covpdb/ and its contents are available for download as flat files of various formats.

## INTRODUCTION

Historically, compounds possessing electrophilic moieties (warheads) with the aptitude to form covalent bonds with disease-associated target proteins were zealously avoided in drug discovery campaigns, due to potential toxicity risks in relation to off-target promiscuity. Early covalent drugs such as aspirin, omeprazole, and beta-lactam antibiotics, were established to act through a covalent bonding mechanism not until long after their market approval (1,2). Conversely, covalent drugs have received tremendous attention in recent years owing to their superior potency, selectivity, and duration of action compared to their noncovalent counterparts (3–5). This paradigm shift is underscored by the fact that up to 30% of all clinically approved drugs act through a covalent bonding mechanism, notably remdesivir, an inhibitor of the severe acute respiratory syndrome coronavirus 2 (SARS-CoV-2) RNA-dependent RNA polymerase (RdRp), serving as one of the very few treatments so far approved for the coronavirus disease 2019 (COVID-19) (6–8). Beyond the clinically approved covalent drugs, there is a further number of targeted covalent inhibitors (TCIs) involved in clinical trials, prominently the orally available small-molecule PF-07321332, which potently and selectively inhibits SARS-CoV-2 main protease (M^pro^) (https://www.clinicaltrials.gov/ct2/show/NCT04756531).

Nowadays, chemoproteomic methods with electrophilic fragment libraries are routinely used to identify TCIs in biochemical and cellular assays (9–12). Moreover, due to rapid advances in protein characterization, there is a plethora of 3D macromolecular structures of covalent protein–ligand (cP–L) complexes that have been resolved using a variety of techniques such as X-ray, NMR, or cryo-EM, and deposited in the Protein Data Bank (PDB) (13). Despite numerous strides made in the field of proteomics, the development of novel TCIs exhibiting high potency and selectivity for a given target protein remains challenging. From a computational standpoint, numerous tools have emerged for the structure-based virtual screening (SBVS) of TCIs. Available covalent molecular docking tools include CovDock (14), GOLD (15), DOCKTITE (16), AutoDock (17), CovalentDock (18), DOCKovalent (19), and DUckCov (20), whereas only LigandScout (21), AncPhore (22), and CSD-CrossMiner (23) have been reported to incorporate covalent pharmacophore modeling.

SBVS endeavors require high quality 3D macromolecular structures as input data. To aid these endeavors, a number of TCI databases have been created from the manual annotation of the published literature. The first such database was Cysteinome (http://www.cysteinome.org), hosting 1,217 pre-reactive ligands, 462 target proteins with targetable Cys residues, and hyperlinks to PDB entries (24). However, the Cysteinome website is no longer accessible. Other TCI databases include: cBinderDB (http://www.rcdd.org.cn/cbinderdb/) describing 1,867 pre-reactive ligands, 555 target proteins with various targetable residues, and 120 PDB structures of cP–L complexes (25); and CovalentInDB (http://cadd.zju.edu.cn/cidb/) describing 4,806 pre-reactive ligands, 298 target proteins with various targetable residues, and less than 280 representative PDB structures of cP–L complexes (26). Since all three above-mentioned databases describe only a small proportion of 3D structures of cP–L complexes, we attempted to fill the gap by adopting a diametrically opposed approach, which consists of the mining of 3D structures of cP–L complexes hosted in the PDB (13). Hence, we created CovPDB, the first database solely dedicated to high-resolution cocrystal structures of biologically relevant cP–L complexes. The 3D structures within CovPDB depict both covalent and noncovalent interactions at the cP–L contact interface, thus providing invaluable insights into structural determinants of molecular recognition processes for the rational design of highly potent and selective TCIs.

## MATERIALS AND METHODS

### Data curation

**Figure 1.**
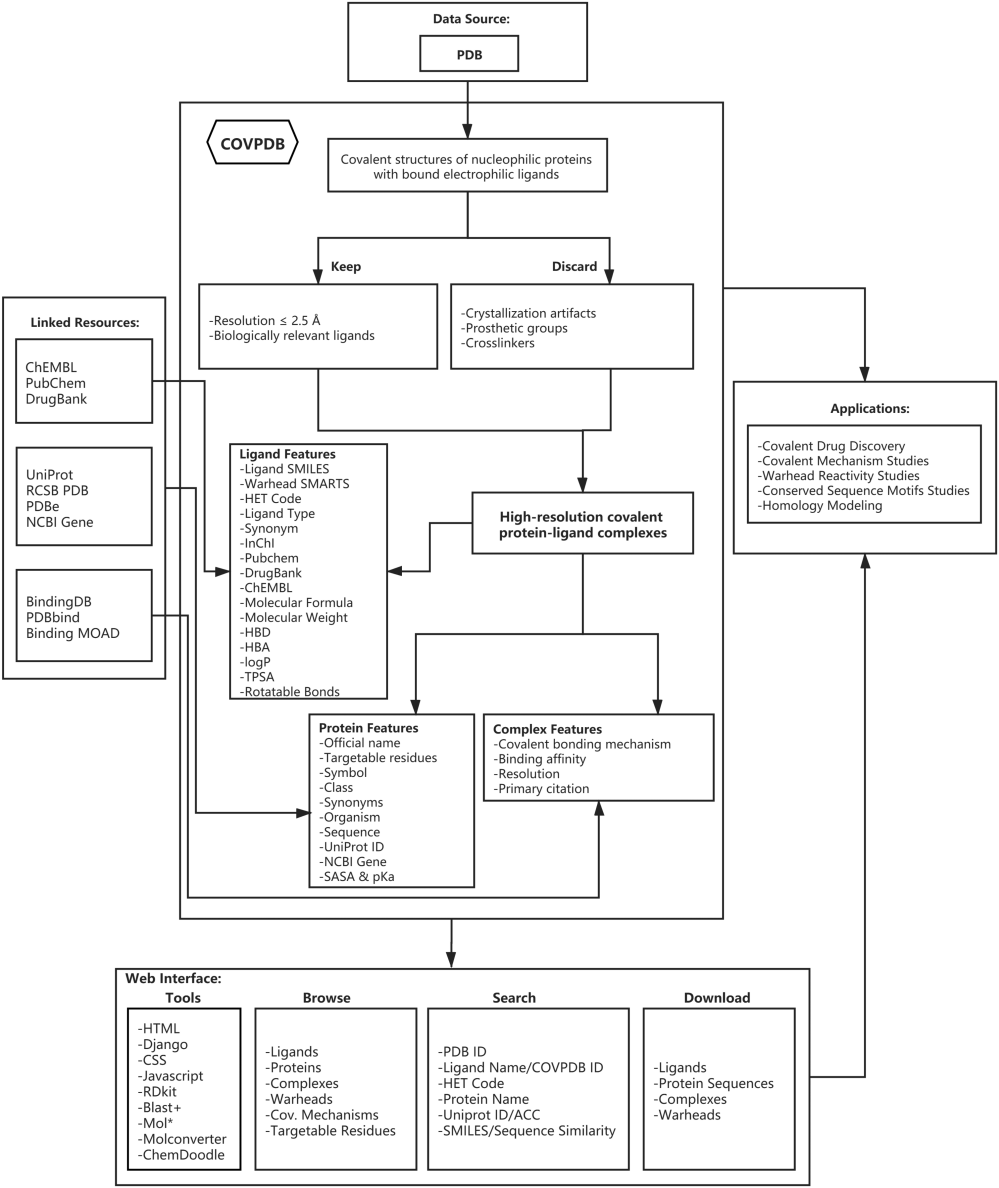
CovPDB flowchart.

This process was carried out in a semi-automated fashion as illustrated in Figure 1. As a starting point, all registered PDB entries were retrieved on 31 August 2020 and parsed with the help of a custom PyMOL (Schrödinger LLC, New York, https://www.schrodinger.com/) Python script for cocrystal covalent structures of target proteins in complex with electrophilic ligands. Because not all PDB structures are suitable for prospective modeling studies, especially those with ambiguous ligand electron densities in the protein binding site, we only kept PDB structures with resolution equal to or better than 2.5 Å. To further refine the collected dataset, the list of artifact PDB ligands (http://zhanglab.ccmb.med.umich.edu/BioLiP/ligand_list) compiled by Zhang *et al.* (27), was used to filter out all retrieved complexes wherein the ligand is biologically irrelevant. Typical artifact ligands include crystallization additives such as glycerol. Additionally, complexes containing covalent cofactors acting as prosthetic groups were discarded (e.g. retinal), and likewise ligands that crosslink two (dis)similar protein chains. The resulting cP–L complexes were visually inspected to ascertain the presence of a covalent bond between interacting pairs. Finally, the chemical structures (SMILES) and warheads (SMARTS) of the pre-reactive electrophilic ligands as well as the covalent bonding mechanisms to their target proteins were expertly manually annotated. This was performed through extraction of structural information from primary citations associated with a given PDB entry. In a few cases with missing primary citations, the features of pre-reactive electrophilic ligands were nonetheless annotated directly from the PDB structures.

For the cP–L complexes of this refined dataset, 3D structure characterization methods, resolutions, and binding affinities were retrieved from PDB (13). Moreover, protein features including official name, symbol, class, synonyms, organism, and sequence were retrieved from PDB (13), UniProt (28), and NCBI Gene (29) records. The acid dissociation constant (p*K*_a_) and solvent accessible surface area (SASA) values of the targetable residues were computationally predicted with PROPKA (30) and FreeSASA (31), respectively. Since the vast majority of the annotated pre-reactive ligands differ in structure to the bound PDB ligands (labeled with PDB HetIDs), each pre-reactive ligand was assigned a unique *‘COVPDB ID*’. Ligand SMILES were used to retrieve PubChem (32), DrugBank (33), and ChEMBL (34) IDs. Additionally, several physicochemical descriptors generally used in druglikeness evaluation of small-molecule ligands were computationally predicted from the ligand SMILES with RDKit (https://www.rdkit.org/).

### Implementation

All curated data was inserted into a PostgreSQL database. The website for CovPDB was implemented using HTML, Django, CSS, and Javascript and supports recent versions of major browsers such as Chrome, Edge, Firefox, Opera and Safari. The Java applets Mol* viewer (35) and ChemDoodle (https://www.chemdoodle.com/) were incorporated into the website for the interactive 3D display of cP–L complexes and ligand structure editing, respectively. Moreover, Molconverter (Marvin 20.18.0, 2020, ChemAxon, https://www.chemaxon.com/) was employed to generate 2D and 3D ligand structures; while RDKit (https://www.rdkit.org/) and NCBI BLAST+ (36) were integrated to the web interface in order to enable ligand fingerprint and protein sequence similarity searches, respectively.

## RESULTS AND DISCUSSION

### Database contents

**Figure 2.**
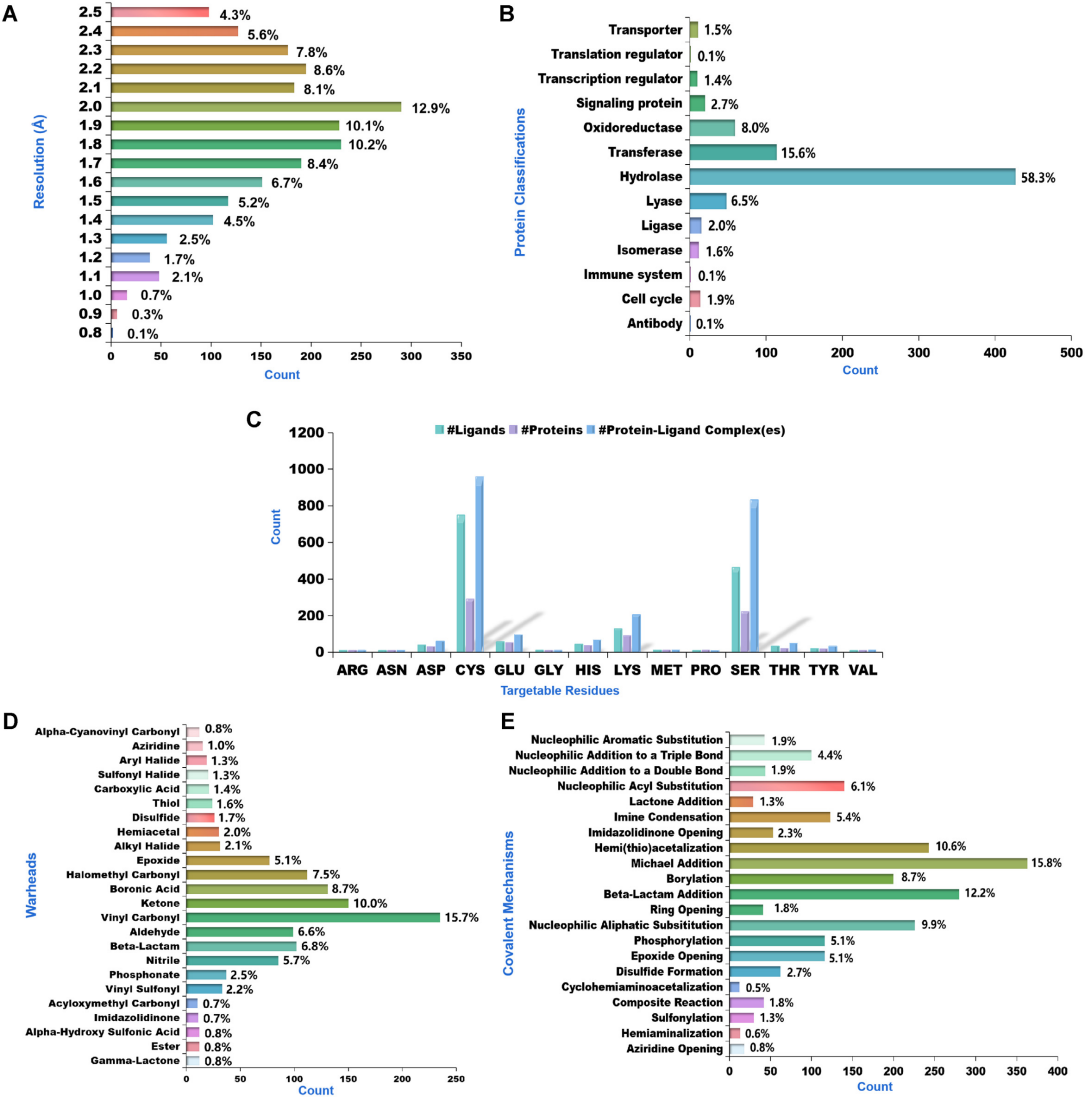
Distribution of CovPDB attributes. (**A**) Distribution of electron density resolution. (**B**) Distribution of protein classes. (**C**) Distribution of targetable residues. (**D**) Distribution of the top 24 warheads. (**E**) Distribution of covalent bonding mechanisms.

Overall, CovPDB contains 1,501 ligands and 733 target proteins, relating to 93 reactive warheads, 14 targetable residues, and 21 covalent mechanisms, as summarized in Table 1. Out of the 2,261 PDB structures, 30 of them contain two different ligands bound to two separate protein chains, one of them (PDB ID: 5TNJ) contains four ligands bound to four separate chains, and the rest contain a single ligand to identical chains, amounting to 2,294 unique cP–L complexes. Only 13 PDB structures were resolved by solution NMR and the rest were resolved by X-ray crystallography with electron density resolution clustered between 1.5 and 2.5 Å (Figure 2A). Compared to CovalentInDB, the newly introduced CovPDB contains twice more proteins and eight times more complexes but thrice less ligands. As such both CovPDB and CovalentInDB are complementary and could be used in an integrated fashion to aid TCI discovery.

**Table 1.** Overview of CovPDB statistics

Attributes	Count
PDB structures	2261
cP–L complexes	2294
Complexes with binding affinities	529
Covalent mechanisms	21
Pre-reactive ligands	1501
Warheads	93
Ligand types	11
Target proteins	733
Protein classes	13
Targetable residue types	14
Source organisms of proteins	261
Literature references	1173

The vast majority of covalently bound ligands within cP–L complexes of the CovPDB are bonded to a single nucleophilic residue (monodentate ligands), 42 are simultaneously bonded to two residues (bidentate ligands), and three are simultaneously bound to three residues (tridentate ligands). It is worth mentioning that the data curation approach adopted in this study did not permit the extraction of cP–L complexes wherein the ligand covalently modifies a cofactor rather than a protein residue, as exemplified by remdesivir, which binds to the RNA cofactor of SARS-CoV-2 RdRp (PDB ID: 7BV2). The protein classification of CovPDB is dominated by hydrolases (Figure 2B). Unsurprisingly, Cys, Ser, and Lys are overly represented among nucleophilic residues of target proteins (Figure 2C). There is a direct correlation between the most represented warhead category (vinyl carbonyl) and the most represented covalent bonding mechanism (Michael addition), as illustrated in Figure 2D and E. Warheads existing in both acyclic and cyclic forms and exhibiting different reactivity profiles to targetable residues were grouped in separate categories, as exemplified by acyclic amides (weakly reactive) and beta-lactams (highly reactive).

### Usage

CovPDB is fully searchable with multiple browsing and search options. From its homepage, six main attribute categories (ligands, proteins, complexes, warheads, covalent mechanisms, and targetable residues) are provided under the ‘Browse’ section as full lists. These lists are interactive and are representative of the attributes distribution. For example, the user can browse the warhead list and obtain the distribution of the five other attributes for each and every one of the 93 warheads, and subsequently retrieve filtered attribute lists for a given warhead (e.g. vinyl carbonyl). From the ‘Search’ category, the user can query the entire database: for proteins by protein name or UniProt ID/ACC; and for ligands by ligand name, COVPDB ID or HET code. Additionally, ligand substructure and similarity (with a threshold) searches can be performed with user-defined SMILES or with a structure sketched via the ChemDoodle (https://www.chemdoodle.com/) editor. Similarly, the user can input a protein sequence to retrieve homologous proteins. And, the results of these two descriptor-based ligand and protein queries can be exported as CSV and TXT files, respectively. For every protein, ligand, or complex entry of the CovPDB, a dedicated card page is provided, which details the experimental and/or computed descriptors of a given attribute (Figure 1). The ligand card contains ligand SMILES, InChI, 2D structure, ligand type, synonyms, PubChem ID, DrugBank ID, ChEMBL ID, molecular weight, molecular weight, octanol/water partition coefficient (log P), H-bond acceptor (HBA), H-bond donor (HBD), rotatable bond count, topological polar surface area (TPSA), and a tabulated list of bound proteins. The protein card contains protein name, synonyms, class, function, sequence, organism, gene symbol, UniProt ID/ACC, Gene ID, PDB ID count, and a tabulated list of bound ligands. The complex card contains PDB ID, resolution, experimental method, PubMed ID (and DOI), tabulated features of the covalent mechanism, and tabulated features of the bound protein and ligand. Additionally, the complex card provides an interactive 3D view of the covalent ligand with the protein binding site, displayed with the Mol* viewer (35) that was developed and utilized by RCSB PDB (13) and PDBe (37) on their websites. All attribute lists and cards are internally linked to one another and externally linked to other online bioinformatic resources.

CovPDB freely provides its contents as flat files for download, namely all ligand structures (SDF format), protein sequences (FASTA format), cP–L complexes (PDB format), and reactive warheads (TXT format). CovPDB contents are amenable to diverse applications in the burgeoning field of rational TCI design. For example, an SBVS endeavor could be initiated from an cP–L complex of interest with a filtered subset of ligand structures that covalently address the nucleophilic residue of the target protein of interest. Additionally, the warhead list could be utilized to filter compound classes of other chemical libraries such as StreptomeDB (38) and ZINC (39), for use in covalent SBVS campaigns. Furthermore, the ligand and cP–L complex datasets could serve as training and benchmark data in warhead reactivity and docking scoring function studies, respectively. On the other hand, conserved sequence motifs and homology modeling studies could be performed with the protein sequences.

## CONCLUSION

Herein, we described the creation of a carefully curated dataset of about 2300 cP–L complexes, 1500 ligands and 750 proteins named as CovPDB (http://www.pharmbioinf.uni-freiburg.de/covpdb/). To the best of our knowledge, this constitutes the largest high-resolution covalent interactome to date. Updates will be made yearly to incorporate newly deposited PDB entries. The user-friendly interface of CovPDB provides a means to intuitively and interactively explore its contents, which are also made available for download as flat files of various formats. Collectively, CovPDB offers valuable insights into the mechanisms by which electrophilic ligands covalently modify nucleophilic residues, as well as the structural determinants of substrate/inhibitor selectivity at a given binding site. It is hoped that these insights will be useful in rationally developing novel TCIs to address unmet medical needs, especially in oncology and infectious diseases, as well as in other diseases.

## DATA AVAILABILITY

CovPDB is freely accessible at http://www.pharmbioinf.uni-freiburg.de/covpdb/ and its contents are available for download as flat files of various formats.
